# CDCA2 Promotes HCC Cells Development via AKT–mTOR Pathway

**DOI:** 10.1155/2022/9912254

**Published:** 2022-12-22

**Authors:** Kai Li, Tingting Fan, Zhongxing Shi, Huijie Jiang

**Affiliations:** ^1^Department of Radiology, The Second Affiliated Hospital of Harbin Medical University, Harbin 150001, China; ^2^Department of Interventional Radiology, Harbin Medical University Cancer Hospital, Harbin 150001, China

## Abstract

**Background:**

Hepatocellular carcinoma (HCC) is a highly aggressive and solid malignancy with a poor prognosis. Cell division cycle associated 2 (CDCA2) is highly expressed in HCC and is considered to be closely related to the prognosis of patients with HCC. In this research, we aimed to investigate the function and potential mechanism of CDCA2 in HCC cells.

**Methods:**

Gain- and loss-of-function experiments were conducted to determine the biological function of CDCA2 in HCC cells. Quantitative reverse transcription-polymerase chain reaction and western blot were utilized to examine the Messenger RNA (mRNA) and protein levels of CDCA2 in HCC cells. The malignant behaviors of HCC cells were analyzed by several biological experiments including cell viability, cell colony formation, and transwell assays. Western blot was also implemented to examine the expression of : AKT, protein kinase B and mTOR, mammalian target of rapamycin (AKT–mTOR) pathway related proteins and Cyclin D1.

**Results:**

A significant increase of CDCA2 was observed in HCC cell lines. Upregulation of CDCA2 resulted in the enhancement of the growth, migration, and invasion of HCC cells. Inversely, depletion of CDCA2 displayed the opposite results. Furthermore, the protein levels of p-AKT, p-mTOR, and Cyclin D1 were elevated with CDCA2 upregulation and reduced with CDCA2 depletion in HCC cells.

**Conclusion:**

Our observations revealed that CDCA2 promoted the malignant development of HCC cells, and AKT–mTOR pathway might involve in the underlying mechanism.

## 1. Introduction

Hepatocellular carcinoma (HCC) is a prevalent malignancy with increasing incidence, which frequently occurs on a background of chronic liver disease [1]. Identified risk factors contain obesity, diabetes, alcohol consumption, and chronic infection with hepatitis B or C viruses, all of which contribute to the growing trend in the incidence of HCC [2]. HCC is featured by an increase in the number of genetically abnormal heterogeneities, leading to tumor progression through uninhibited cell proliferation and higher potential for invasion and metastasis [3]. Surgical resection and transplantation are the best treatment options for early-stage HCC, whereas the curative effect of advanced HCC is far from ideal [4]. Thus, therapeutic breakthroughs are still needed.

The emergence of molecularly targeted therapies has attracted extensive attention, which interfere with specific molecules to suppress tumor growth, development, and metastasis [5]. Over the past years, development of the molecular cell biology has helped us gain a better understanding of the detailed molecular mechanisms underlying tumorigenesis. Furthermore, this gave opportunities to identify novel molecular-targeted agents, which suppress molecular abnormalities, as hopeful therapeutic strategy for cancer [6]. To date, several targeted agents including sorafenib, regorafenib, and lenvatinib have been approved to improve survival in systemic treatment of HCC [7–9]. Since the above-mentioned targeted drugs have some side effects, exploring more effective targets is necessary for targeted therapy of HCC.

Cell division cycle associated 2 (CDCA2), also named recruits protein phosphatase 1 (PP1) onto mitotic chromatin at anaphase, is a nuclear protein that binds to PP1 and regulates cell cycle [10]. CDCA2 is responsible for PP1 targeting chromatin in anaphase, resulting in H3 dephosphorylation and controlling cell growth *in vitro* [11–13]. Moreover, CDCA2 was found to regulate the expression of PP1 *γ*-dependent essential deoxyribonucleic acid (DNA) damage response [13, 14]. A growing body of evidence has revealed that CDCA2 is a crucial factor in cancer development. For example, a marked augmentation of CDCA2 is a common event for oral cancer, and its overexpression blocked G1 arrest by suppressing cyclin-dependent kinase inhibitors and triggering apoptosis [15, 16]. In 2022, Tang et al. investigated the expression pattern and the prognostic value of CDCA2 in HCC based on the data from The Cancer Genome Atlas database (TCGA-LIHC) using bioinformatics methods. They revealed that CDCA2 is an independent prognostic biomarker in HCC, and enhanced expression of CDCA2 is correlated with the up-regulation of immune checkpoints [17]. However, the exact role of CDCA2 on the malignant growth of HCC cell has not been studied. Herein, we attempted to assess the ability of HCC cell line to grow, migrate, and invade depending on CDCA2 expression levels using biological experiments and investigate the potential mechanism.

## 2. Materials and Methods

### 2.1. Cell Culture and Treatment

Human HCC cells (HepG2 and Huh7) and normal control cell L-02 were gained from Cell Bank of the Chinese Academy of Sciences (Shanghai, China). The cells were grown in an RPMI-1640 medium appended with 10% heat-inactivated fetal bovine serum including antibiotics. Cells were grown in an atmosphere of 5% CO_2_ at 37°C. Short interfering ribonucleic acids (siRNAs) specifically targeting CDCA2 were applied for construction of CDCA2-depleted HCC cells, and scramble siRNA acted as a negative control (si-con). Expression vector containing CDCA2 was designed to construct CDCA2-overexpressed HCC cells. All small molecules were acquired from Sangon Biotech (Shanghai, China). Transient transfection was implemented with the assistance of Lipofectamine 2000 in steps with supplier's direction. The following experiments were conducted after 48 hours of transfection.

### 2.2. Quantitative Polymerase Chain Reaction Analysis

After treatment, the total RNA in cells was isolated by TRIzol in steps with supplier's direction. Single-stranded complementary DNA was synthesized with the assistance of a PrimeScript RT Reagent Kit, TaKara, Japan. Reverse transcription polymerase chain reaction (RT-PCR) was carried out by utilizing an SYBR Green PCR Kit, QIAGEN, Germany following the supplier's direction. The relative expression of CDCA2 was estimated by 2^−*ΔΔ*CT^ method. glyceraldehyde-3-phosphate dehydrogenase (GAPDH) was regarded as a standard control. The primers used in this study were listed as below:

CDCA2:

F: 5′-GAGGCAGGAAAAGAGTCCGAGA-3′,

R: 5′-CTCCGACGTTTGGAGGACAACA-3′;

GAPDH:

F: 5′-GTCTCCTCTGACTTCAACAGCG-3′,

R: 5′-ACCACCCTGTTGCTGTAGCCAA-3′.

### 2.3. Western Blotting

The treated cells were rinsed by pre-cooling phosphate buffered saline (PBS) and collected by gentle scraping. Cell lysates were obtained by using Radio Immunoprecipitation Assay, a common cell lysate (RIPA) lysis including protease inhibitor. Equal amount of cell lysates (quantified by a bicinchoninic acid, A common method for determination of protein concentration (BCA) method) were resolved on dodecyl sulfate, sodium salt (SDS)-Polyacrylamide gel electrophoresis, a protein imprinting technique (SDS-PAGE) and transferred to a polyvinylidene fluoride, a hydrophobic polymer membrane for protein imprinting (PVDF) membrane and subjected to immunoblot with the primary antibodies for CDCA2, AKT, p-AKT, mTOR, p-mTOR, Cyclin D1, and GAPDH in blocking buffer, and subsequently, the membranes were incubated with the appropriate secondary antibody for 2.5 hours. Detection was performed by using enhanced chemiluminescence, and the gray values of the specific bands were quantified by the ImageJ software.

### 2.4. Cell Viability Assay

A Cell Counting Kit-8 (CCK-8), Beyotime, Beijing, CHINA reagent was applied to examine cell viability. A total of 5 × 10^3^ cells placed in a 96-well microplate were maintained at 37°C for appointed time. The viability of cells was calculated every 24 hours by adding 10% CCK-8 reagent in steps with the supplier's instruction.

### 2.5. Cell Colony Formation Assay

A total of 500 transfected cells were placed into a 60 mm dish. The dish was incubated at 37°C, and the culture was terminated when visible colonies appeared. After being washed by PBS, colonies were fixed by paraformaldehyde (4%) and stained with crystal violet solution (1%) for 30 minutes. The number of the colonies was counted manually.

### 2.6. Transwell Assay

Cell invasion ability was estimated with the support of transwell chambers. A total of 1 × 10^5^ cells were placed in the top of a transwell filter, which pre-coated with Matrigel and loaded with 100 *μ*L serum-free medium. In the lower chamber, fresh complete medium was added. After 24 hours of incubation, the invaded cells at the base of the transwell filter were stained. The number of cells was counted in 5 random fields under a light microscope (magnification ×200). The migration assay was the same as this, excluding that the top chamber was pre-coated with Matrigel.

### 2.7. Statistical Analysis

The experimental values were expressed as mean ± SD acquired from appropriate number of independent experiments conducted in triplicates. Statistical significance was evaluated by utilizing SPSS 22.0 and GraphPad Prism 7.0. Student's *t*-test was used to analyze data involving direct difference of an experiment group with a control group. Analysis of Variance, Method for significance test of mean difference between two or more samples (ANOVA) followed by Bonferroni's *post hoc* test was applied to analyze the comparison among multiple experimental groups. For all statistical tests, *p*-value less than 0.05 was accepted for statistical significance.

## 3. Results

### 3.1. CDCA2 Promoted the Viability and Colony Formation Ability of HCC Cells

**Figure 1:**
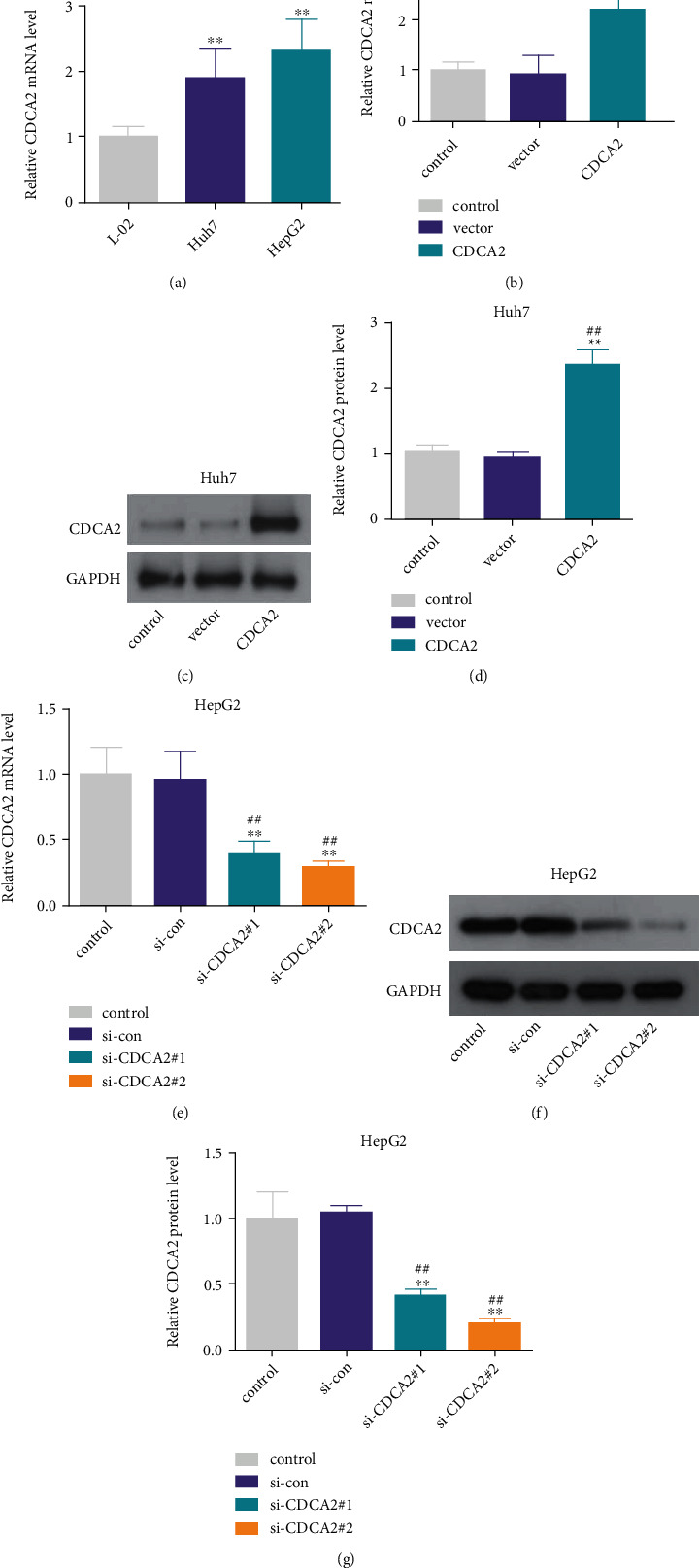
Knockdown or overexpression of cell division cycle associated 2 (CDCA2) in hepatocellular carcinoma (HCC) cells. (a) The expression of CDCA2 was higher in HCC cells than that in L-02 cells, *p* < 0.01 versus L-02. (b)–(d) CDCA2 expression was obviously elevated at mRNA and protein levels with pcDNA3.1-CDCA2 stimulation. ∗∗*p* < 0.01 versus control and ^##^*p* < 0.01 versus vector. (e)–(g) si-CDCA2#1 or si-CDCA2#2 treatment obviously suppressed CDCA2 expression than the control and si-con. ∗∗*p* < 0.01 versus control and ##*p* < 0.01 versus si-con.

**Figure 2:**
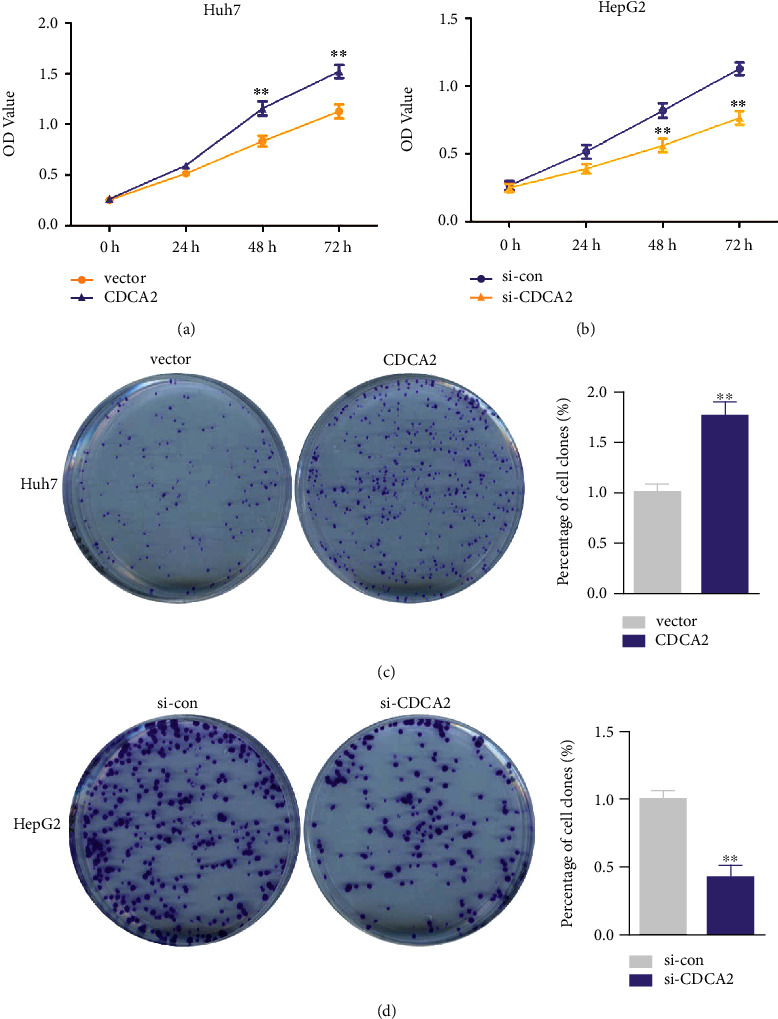
Estimation of hepatocellular carcinoma cells growth ability with cell division cycle associated 2 (CDCA2) abnormal expression. (a) The OD_450_ values in Huh7 cells were significantly increased with CDCA2 upregulation. (b) Knockdown of CDCA2 decreased the OD_450_ values in HepG2 cells. (c) The colonies number in Huh7 cells was obviously elevated with CDCA2 overexpression. (d) CDCA2 deletion eliminated the increasing trend of HepG2 cells clone number. ∗∗*p* < 0.01 versus vector or si-con.

To identify whether CDCA2 has the potential to drive HCC cells growth, the effect of CDCA2 on viability and colony formation ability of HCC cells was measured by gain- and loss-of-function experiments in Huh7 and HepG2 cells. First, we measured the expression of CDCA2 in HCC cell lines (Huh7 and HepG2), and the human hepatocyte L-02 was served as a control. As presented in Figure 1(a), RT-PCR data revealed that the expression level of CDCA2 was elevated in HCC cell lines when compared with that in L-02 cells. We choose Huh7 for overexpression and HepG2 for silencing experiments since that the expression of CDCA2 was higher in HepG2 than that in Huh7. As displayed in Figures 1(b), 1(c), and 1(d), pcDNA3.1-CDCA2 significantly increased CDCA2 expression in Huh7 cells at both Messenger RNA (mRNA) and protein levels. The data from Figures 1(e), 1(f), and1(g) exhibited that CDCA2 mRNA and protein expression levels were notably reduced when treated by si-CDCA2#1 or si-CDCA2#2, and si-CDCA2#2 with a higher interference efficiency was selected for subsequently loss-of-function experiment. From the cell viability assay and colony formation assay, we discovered that CDCA2 upregulation increased the viability and colony formation number of Huh7 cells than the control (Figures 2(a) and 2(c)). In contrast, depletion of CDCA2 notably decreased the viability and colony formation number of HepG2 cells (Figures 2(b) and 2(d)). These data illustrated that CDCA2 may accelerate HCC cells growth *in vitro*.

### 3.2. CDCA2 Facilitated the Movement of HCC Cells

**Figure 3:**
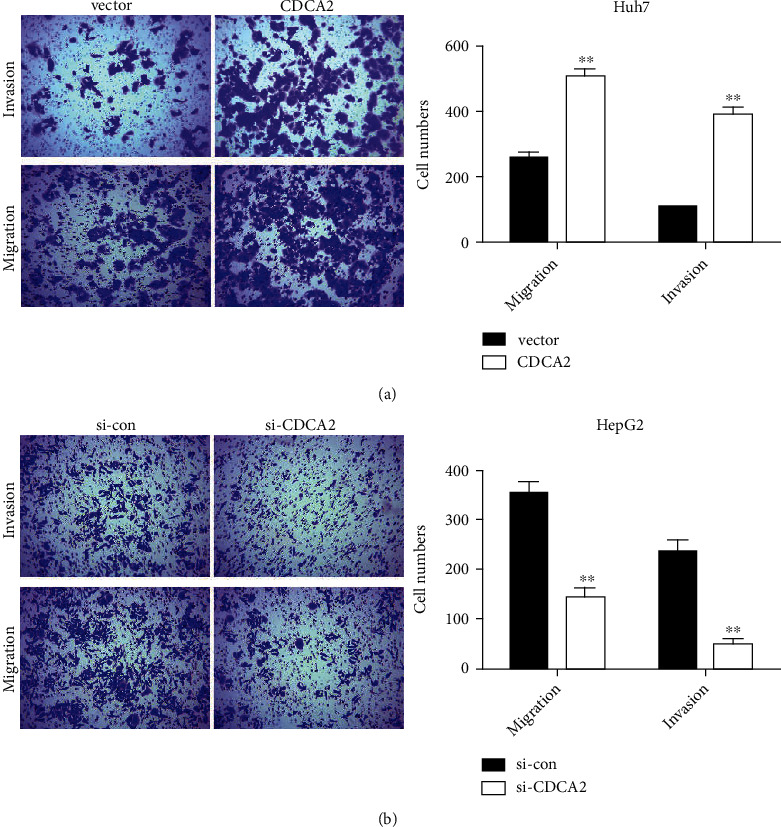
Aberrant expression of cell division cycle associated 2 (CDCA2) affected the movement of hepatocellular carcinoma cells. (a) The invasion and migration abilities of Huh7 cells were obviously enhanced with CDCA2 overexpression. (b) Silencing of CDCA2 repressed the movement ability of HepG2. ∗∗*p* < 0.01 versus vector or si-con.

Subsequently, to gain an insight into the role of CDCA2 in cell invasion and migration, transwell chamber was applied. The results indicated that CDCA2 overexpression notably strengthened the movement potential of Huh7 cells than the control group, as larger numbers of migrated and invaded cells were observed in the experimental group (Figure 3(a)). Inversely, exhaustion of CDCA2 significantly repressed the invasive and migratory properties of HepG2 cells (Figure 3(b)). These data revealed that CDCA2 may promote the invasion and migration of HCC cells *in vitro*.

### 3.3. High Expression of CDCA2 Promoted Malignant Features of HCC Cells Partly by Activation of AKT/mTOR Pathway and Cyclin D1

**Figure 4:**
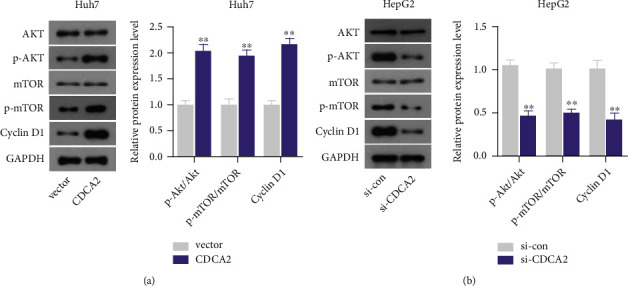
Evaluate the effect of cell division cycle associated 2 (CDCA2) on the expression of key factors. (a) Upregulation of CDCA2 increased the expression of p-AKT, p-mTOR, and Cyclin D1. (b) Exhaustion of CDCA2 inhibited the expression of p-AKT, p-mTOR, and Cyclin D1. ∗∗*p* < 0.01 versus vector or si-con.

To gain an insight into the mechanisms by which CDCA2 promoted the malignant properties of HCC cells, we detected AKT, p-AKT, mTOR, p-mTOR, and Cyclin D1 expression in HCC cells. We observed that CDCA2 upregulation increased the levels of p-AKT, p-mTOR, and Cyclin D1 in Huh7 cells compared with the vector group (Figure 4(a)), whereas the expression of AKT and mTOR in Huh7 cells did not present significant difference between CDCA2 and vector groups (Figure 4(a)). In contrast, depletion of CDCA2 repressed the levels of p-AKT, p-mTOR, and Cyclin D1 in HepG2 cells (Figure 4(b)). However, knockdown of CDCA2 has no significant effect on AKT and mTOR expression (Figure 4(b)).

## 4. Discussion

The present study revealed that depletion of CDCA2 *in vitro* suppressed the cellular growth, invasion, and migration, whereas overexpression of CDCA2 promoted the growth and motility of HCC cells. Of note, these effects were at least partially mediated by the AKT/mTOR pathway and Cyclin D1 *in vitro*. These findings insinuated that CDCA2 might play an oncogenic role in the cellular growth and mobility in HCC cells.

CDCA2 was first authenticated as a PP1 binding protein by Trinkle-Mulcahy et al. [12]. Later, it was found that CDCA2 was closely related to DNA damage and cell cycle, and the role of CDCA2 in cancers has also received more and more attention [12, 18, 19]. For example, it can contribute to lung cancer cells growth and predict worse prognosis in lung cancer patients [20]. In addition, it suppressed apoptosis and accelerated cell growth in prostate cancer by hypoxia inducible factor-1,it is a transcription factor widely existing in mammals and human body under hypoxia conditions, and a key factor in response to hypoxia stress (HIF-1*α*) pathway [10]. Besides, CDCA2 high expression may be closely related to oral squamous cell carcinoma, a types of head and neck squamous cell carcinoma (OSCC) development by blocking cell cycle arrest and apoptosis [16]. In addition to reports, which revealed that overexpression of CDCA2 is a common event in lung, prostate, and oral cancers, Tang et al. reported that CDCA2 was increased in HCC as well based on analyses of data from TCGA [17]. Furthermore, they suggested that CDCA2 had a high diagnostic power and was associated with poor survival for HCC [17]. On the basis of these reports, we investigated the effects of CDCA2 on the malignant behaviors of HCC cells using biological experiments in the present study. We found that high expression level of CDCA2 resulted in the high proliferation and invasion properties of HCC cells. Anteriorly, CDCA2 was reported to be a member of a group of proteins that associated with known cell cycle related protein, such as CDCA1, CDCA3, and CDCA4–8 [11], which indicating that CDCA2 has an important role in the cell cycle. In the present study, we found that CDCA2 upregulation resulted in the increasing trend of Cyclin D1 protein expression, which indicating that CDCA2 might promote the proliferation of HCC cells through upregulation of Cyclin D1 protein expression.

mTOR is a serine/threonine protein kinase, whose abnormal activation is very general in malignant cells, resulting in tumor initiation and progression [21]. AKT, an upstream factor of mTOR, activates mTOR through phosphorylation of TSC1/TSC2 complex [22]. Moreover, mTOR also mediates phosphorylation of AKT at Ser473, regulating mRNA translation and cell survival [23]. In the past few years, much has been learned about the molecular mechanisms by which the AKT–mTOR pathway regulates cell proliferation, growth, cell cycle, survival, and protein synthesis [24, 25]. In steps with its physiological role, the AKT–mTOR pathway has been revealed to be hyperactivated in numerous tumors [26]. For example, activation of AKT–mTOR pathway contributes to the progression of lung cancer, breast cancer, endometrial cancer, and HCC [27–30]. Using gene set enrichment analysis, Tang et al. demonstrated that CDCA2 may be involved in mTOR pathway [17]. Consistently, by biological experiments, we discovered that overexpression of CDCA2 triggered the increasing of phosphorylation of mTOR and AKT in the present study. It is indicated that CDCA2 accelerated the malignant behaviors of HCC cells might partially through activating AKT–mTOR pathway.

Of note, several limitations in our study should be revealed. First, AKT–mTOR pathway is only one of many pathways related to CDCA2/HCC, and more related pathways will be explored in the future study. Second, more indicators related to cell cycle should be detected in future. Third, the results will be verified in the next stage of animal experiments.

Collectively, our data indicated that overexpression of CDCA2 facilitated the growth and mobility of HCC cells partly through controlling AKT–mTOR pathway, providing evidence for CDCA2 as a biomarker and a potential target molecule for the treatment of HCC.

## Data Availability

The data used to support the findings of this study are included within the article.
